# Comparative Epidemiology of *Salmonella enterica *Serovers Paratyphi A and Typhi Causing Enteric Fever, Bangladesh, 2018–2020 

**DOI:** 10.3201/eid3110.241601

**Published:** 2025-10

**Authors:** Sadia Isfat Ara Rahman, Md Golam Firoj, Se Eun Park, Farhana Khanam, Suneth Agampodi, Kassa Haile, Edilawit Mesfin, Faisal Ahmmed, Md Taufiqul Islam, Ashraful Islam Khan, Fahima Chowdhury, Afroza Akter, Martin Bundi Mwebia, Justin Im, Natasha Y. Rickett, Cecilia Kathure Mbae, Asma Binte Aziz, Beatrice Ongadi, Moses Mwangi, Benjamin Ngugi, Meseret Gebre Behute, Kelvin Kering, Suman Kanungo, Xinxue Liu, Deok Ryun Kim, Andrew J. Pollard, K Zaman, Samuel Kariuki, Firdausi Qadri, John D. Clemens

**Affiliations:** icddr,b, Dhaka, Bangladesh (S.I.A. Rahman, M.G. Firoj, F. Khanam, F. Ahmmed, M.T. Islam, A.I. Khan, F. Chowdhury, A. Akter, K. Zaman, F. Qadri, J.D. Clemens); Yonsei University Graduate School of Public Health, Seoul, South Korea (S.E. Park); International Vaccine Institute, Seoul (S.E. Park, S. Agampodi, N.Y. Rickett, A.B. Aziz, M.G. Behute, D.R. Kim, J.D. Clemens); Armauer Hansen Research Institute, Addis Ababa, Ethiopia (K. Haile, E. Mesfin); Kenya Medical Research Institute, Nairobi, Kenya (M.B. Mwebia, C.K. Mbae, B. Ongadi, M. Mwangi, B. Ngugi, K. Kering, S. Kariuki); RIGHT Foundation, Seoul (J. Im); ICMR, National Institute of Cholera and Enteric Diseases, Kolkata, India (S. Kanungo); University of Oxford, Oxford, UK (X. Liu, A.J. Pollard); NIHR Oxford Biomedical Research Centre and Oxford University Hospitals NHS Foundation Trust, Oxford (A.J. Pollard); UCLA Fielding School of Public Health, Los Angeles, California, USA (J.D. Clemens); Korea University Vaccine Innovation Center, Seoul (J.D. Clemens)

**Keywords:** Salmonella, bacteria, enteric infections, epidemiology, antimicrobial resistance, incidence, Bangladesh

## Abstract

Enteric fever remains a public health challenge. We analyzed data from a cluster-randomized Vi-tetanus toxoid conjugate vaccine trial to compare the epidemiology between *Salmonella enterica* serovars Paratyphi A, which causes paratyphoid fever, and Typhi, which causes typhoid fever. The overall incidence rate of paratyphoid fever was 27 (95% CI 23–32)/100,000 person-years (PY) and of typhoid fever was 216 (95% CI 198–236)/100,000 PY. We observed the highest incidence for both diseases in children 2–4 years of age: 72 (95% CI 41–117)/100,000 PY for paratyphoid and 887 (95% CI 715–1,088)/100,000 PY for typhoid. Lack of private toilets and safe drinking water were associated with both diseases. Prevalence of multidrug resistance was significantly higher in *Salmonella* Typhi (20.2%) than in *Salmonella* Paratyphi A (0.8%) (p<0.001). Our data suggest that integrated control measures targeting water, sanitation, and hygiene measures and bivalent vaccine targeting both pathogens are promising strategies to control both diseases.

Enteric fever is caused by *Salmonella enterica* serovars Paratyphi A, B, and C, which cause paratyphoid fever, and Typhi, which causes typhoid fever. Globally, an estimated 9.3 million cases and 107,459 deaths related to typhoid and paratyphoid fevers occurred in 2021; they especially affected children living in low- and middle-income countries (*1*). Although *Salmonella* Typhi played a major role, *Salmonella* Paratyphi A constituted 23.3% of enteric fever cases in South Asia countries (*1*). Preventing enteric fever by improving water, sanitation, and hygiene (WASH) infrastructure and practices remains challenging where resources are constrained, placing great reliance on antimicrobial therapy. Increasing multidrug resistance (MDR), defined as resistance to former first-line drugs including ampicillin, chloramphenicol, and trimethoprim/sulfamethoxazole (cotrimoxazole), in *Salmonella* Typhi is hampering successful therapy (*2*–*5*). However, previous studies show that MDR rates declined before the emergence of fluoroquinolone-nonsusceptible *Salmonella* Paratyphi A and *Salmonella* Typhi strains that render fluoroquinolone less suitable as first-line therapy prescribed by clinicians (*6*–*11*). Furthermore, the increase in azithromycin resistance in some settings is worrisome (*12*,*13*). Given the overlapping clinical features of *Salmonella* Paratyphi A infections, laboratory diagnostics are essential for distinguishing between the pathogens to guide appropriate antimicrobial therapy. Because antimicrobial resistance (AMR) makes treatment more difficult, vaccines and WASH interventions play a vital role in prevention.

World Health Organization (WHO)–approved typhoid conjugate vaccines (TCVs) are available (*14*–*18*), and bivalent vaccines targeting both *Salmonella* Paratyphi A and *Salmonella* Typhi are in development (*19*). In this study, we analyzed data from a cluster randomized trial of Vi-tetanus (Vi-TT) toxoid conjugate vaccine, based on the Vi capsular polysaccharide of *Salmonella* Typhi, in urban Dhaka, Bangladesh (*20*), to assess epidemiologic features of enteric fever. We conducted this study as part of the original vaccine trial study (protocol no. PR-17115), which received ethical approval from the research review committee and the ethical review committee of icddr,b in Dhaka. No additional ethics submission was required. We obtained informed written consent from legal guardians of child participants and from adult participants.

## Methods

### Study Design and Site

**Figure 1:**
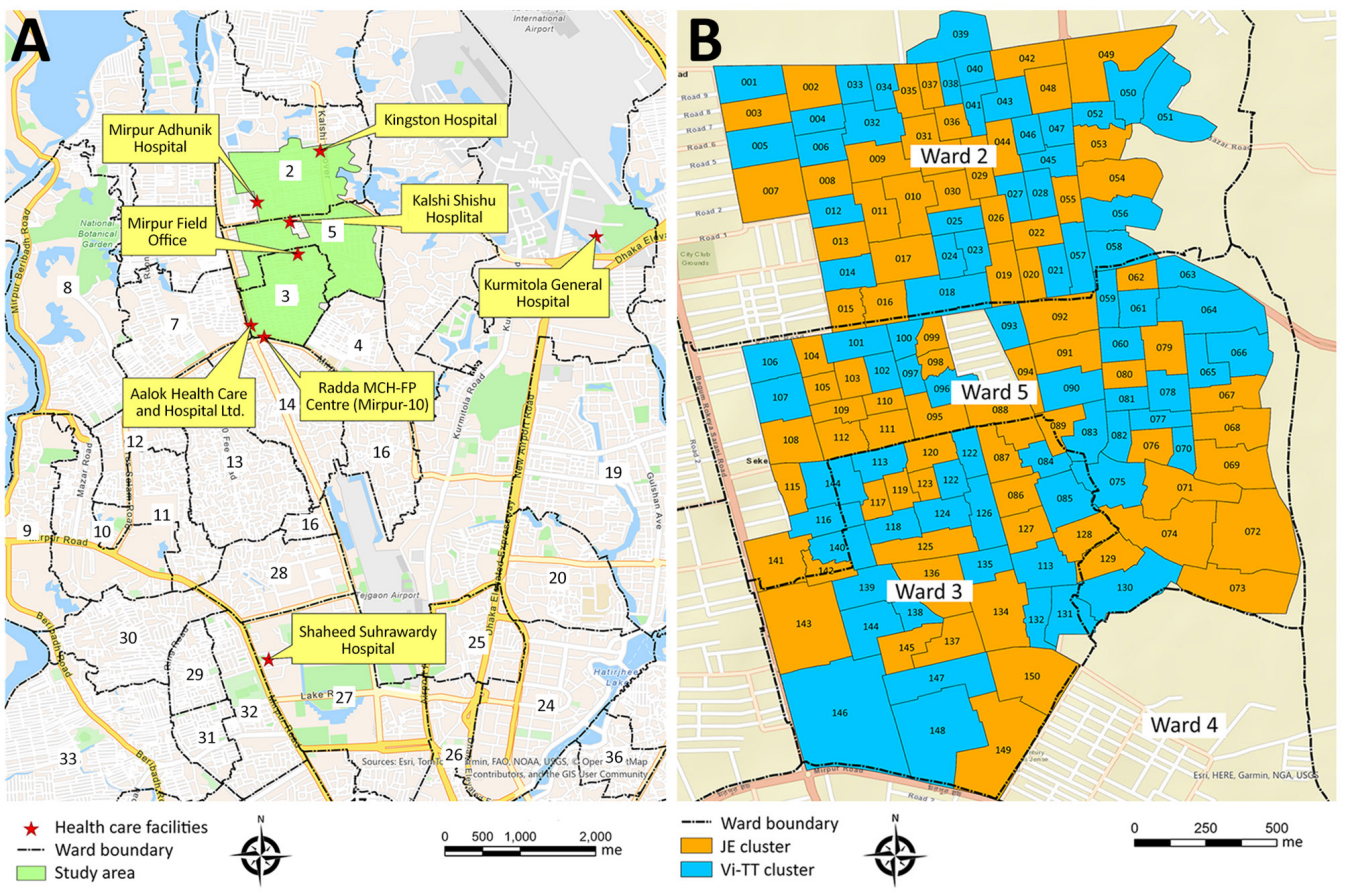
Study area for comparative epidemiologic study of *Salmonella enterica *Serovers A and Typhi causing enteric fever, Dhaka, Bangladesh, 2018–2020. A) Study areas (green) and sentinel surveillance healthcare facilities (red stars). B) Clusters within the study area in Vi-TT effectiveness trial; orange shows JE cluster and blue shows Vi-TT cluster. Maps were generated using OpenStreetMap (http://www.OpenStreetMap.org), Esri World Street Map (http://goto.arcgisonline.com/maps/World_Street_Map), and Dhaka North City Corporation Administration Map (https://dncc.portal.gov.bd/site/page/52cb2e96-9af8-4e8c-ab22-b542e0cc373c*)*; maps were digitized and updated by icddr,b. JE, Japanese encephalitis vaccine; Vi-TT, Vi-tetanus toxoid conjugate vaccine.

We used the data from a participant- and observer-blind, cluster-randomized, controlled trial of Vi-TT vaccine effectiveness study (*20*), which was conducted in a densely populated urban area of wards 2, 3, and 5 of Mirpur in Dhaka (Figure 1). Vi-TT was the study vaccine, and the Japanese encephalitis (JE) vaccine was the control vaccine (*21*). The study area was divided into 150 geographic clusters randomly assigned to the Vi-TT or JE vaccine arms with a 1:1 ratio (*20*). A baseline census was conducted during February 14–March 25, 2018, to enumerate the entire population in this study area. The census was subsequently updated at 6-month intervals to capture all births, deaths, and migrations. Individual-level and household-level demographic and socioeconomic data, household geopositioning, and WASH data were collected at each census (*20*).

### Passive Surveillance for Enteric Fever

We conducted passive surveillance during April 26, 2018–March 15, 2020, in the 8 main healthcare facilities (HCFs) serving the study population: Mirpur Field Office, Shaheed Suhrawardy Hospital, Radda MCH-FP Centre (Mirpur-10), Mirpur Adhunik Hospital, Kalshi Shishu Hospital, Kurmitola General Hospital, Kingston Hospital, and Aalok Health Care and Hospital Ltd (Figure 1) (*20*). We enrolled participants of all age groups living in the surveillance catchment area who had a history of fever (>48 hours) or objective fever (axillary temperature >38.0°C) at the time of HCF visit after they or a parent provided written informed consent. We collected blood specimens (3 mL from participants <17 years of age and 5 mL from those >17 years of age) and clinical data upon enrollment. At the time they sought clinical care, we confirmed patient identity either through household identity cards distributed during the census or via census on electronic tablets when identity cards were unavailable.

### Laboratory Analysis

We performed microbiologic culture of blood samples using a standard automated BacT/ALERT method (*22*). When we detected a positive signal from the automated culture machine, we took subcultures on MacConkey agar plates. After overnight incubation at 37°C, we inoculated non–lactose fermenting colonies on Kligler’s Iron Agar (KIA), Motile Indole Urea (MIU), and citrate tubes for biochemical testing to identify *Salmonella* spp.; we serotyped specimens with *Salmonella*-specific O and flagellar H antiserum (Denka Sieken, https://www.denka.co.jp) to confirm *Salmonella* Paratyphi A, B, and C and *Salmonella* Typhi. We performed antibiotic susceptibility tests (ASTs) for ampicillin, chloramphenicol, trimethoprim/sulfamethoxazole, amoxiclav, azithromycin, cefixime, ceftriaxone, gentamycin, meropenem, ciprofloxacin, and nalidixic acid using the disk diffusion method on antibiotic disks and determined resistance profile in accordance with Clinical and Laboratory Standards Institute guidelines (*23*).

### Definitions

We defined a treatment visit for paratyphoid or typhoid fever as a visit for fever in which the enrolled patient submitted a blood culture positive for *Salmonella* Paratyphi A or *Salmonella* Typhi. We concatenated visits for fever in which the onset of symptoms occurred within 14 days of discharge from the previous visit into the same episode. We set the starting date of follow-up as the median date (April 30, 2018) of the baseline vaccination campaign (April 15–May 15, 2018) for unvaccinated patients and the date of actual vaccination for vaccinated patients. For any persons entering our surveillance area (e.g., new births, migration into the study area) after the baseline vaccination campaign, their follow-up period started at the date of birth or date of entry.

### Strategies for Analyses

We followed each participant for up to 23 months (April 15, 2018–March 15, 2020) to track the *Salmonella* Paratyphi A and *Salmonella* Typhi episodes. We considered participants residing in clusters for both Vi-TT and JE arms for analysis of *Salmonella* Paratyphi A episodes but considered only those in the JE arm for analysis of *Salmonella* Typhi episodes. We conducted analyses using both closed cohort and dynamic cohort approaches. We defined the closed cohort as participants who were included in the baseline census or were present at the median date (April 30, 2018) of the baseline vaccination or both. We conducted the closed cohort analysis to evaluate the incidence rates and to measure the associations of disease occurrence with baseline features including sociodemographic and WASH characteristics. We considered only the first episodes for incidence calculation. We measured age-stratified incidence by considering the age of each patient at baseline.

The dynamic cohort referred to the entire population throughout the study period, including those added through birth, immigration after the baseline census, and reentry into the study area after earlier emigration. We conducted dynamic-cohort analysis to provide the best depiction of the disease burden with seasonality, incidence rate (overall and age-stratified), clinical features, and AMR pattern. We calculated seasonality by exploring the monthly incidence of the diseases. For incidence calculation, we considered all episodes detected after starting the follow-up. For age-specific incidence, we used age at follow-up, expressed as age intervals; the numerator was episodes occurring in the population as it passed through the age interval of interest during follow-up, and the denominator was calendar person-time as the population passed through the age interval during follow-up. We conducted descriptive comparative analyses of the clinical features and AMR patterns using the episodes. We did not include follow-up of participants in both cohorts after the dates of death or migration out from the study area or the end of the surveillance (March 15, 2020).

### Statistical Analyses

We estimated incidence rates as the total number of *Salmonella* Paratyphi A or *Salmonella* Typhi episodes (numerator) divided by the corresponding person-time of follow-up (denominator). In incidence estimation by age or month for seasonality, we considered the numerator as the total episodes from that age or calendar-time period of interest, and the denominator was the total calendar person-time at that age or calendar-time period of interest. We calculated 95% CIs of the incidence rates using the Byar method (*24*). To identify factors independently associated with the disease, we conducted time-to-event analysis using Cox proportional hazards model, adjusting for age, sex, and design effect. We assessed intracluster correlation (ICC) to investigate the clustering effects on paratyphoid and typhoid fever incidence at the levels of the randomized clusters and households. We calculated ICCs using a generalized linear mixed model under maximum-likelihood estimation via Laplace approximation with the Poisson model as the within-subjects probability model under the repeatability settings (*25*).

We used χ^2^ test to measure the associations between categorical variables and Fisher exact test for small (<5) cell frequencies. We assessed continuous variables with *t*-test or nonparametric Mann-Whitney U test if the data violated distributional assumptions. We considered a 2-tailed test at a 5% level of significance for all statistical analysis. We performed analysis with R statistical software version 4.3.3 (The R Project for Statistical Computing, https://www.r-project.org) using the epiR version 2.0.78 package to estimate the incidence rate and iccCounts for the calculation of ICC.

## Results

### Study Population and Episodes of Paratyphoid and Typhoid Fever 

We enumerated a total of 205,760 participants during the baseline census, of which 102,698 were from the Vi-TT arm and 103,062 were from the JE arm. For the closed cohort, we considered 206,065 participants, 205,760 from the baseline population and 305 new births before the median date of the baseline vaccination. After the subsequent censuses during the study period, we added 120,729 participants, 6,157 from birth and 114,572 from immigration. As a result, we considered a total of 326,794 participants for the dynamic cohort.

**Figure 2:**
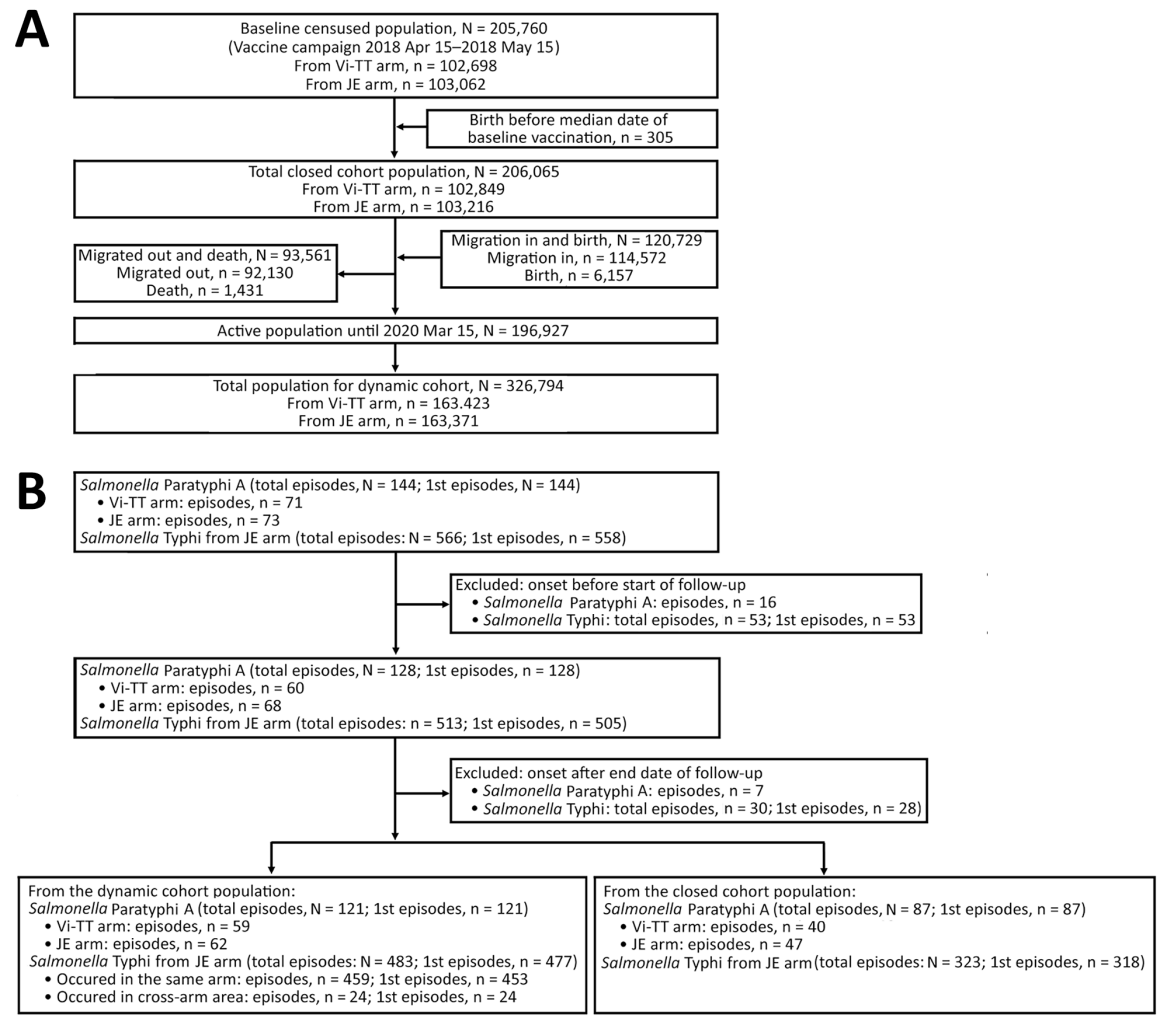
Selection flowcharts of the population (A) and case consort (B) in study of comparative epidemiology of *Salmonella enterica *Serovers Paratyphi A and Typhi causing enteric fever, Bangladesh, 2018–2020. JE, Japanese encephalitis vaccine; Vi-TT, Vi-tetanus toxoid conjugate vaccine.

Throughout the surveillance of the dynamic cohort, we found 144 episodes of *Salmonella* Paratyphi A from both the Vi-TT and JE arms and 566 episodes of *Salmonella* Typhi only from the JE arm. After excluding the episodes that occurred between the end of the study period and the start of follow-up, the total number of *Salmonella* Paratyphi A cases reported from the dynamic cohort was 121 and the total of *Salmonella* Typhi episodes was 483. Of the 483 episodes of *Salmonella* Typhi, 6 were recurrent, whereas all *Salmonella* Paratyphi A episodes were first episodes. In the closed cohort, the total number of first episodes of *Salmonella* Paratyphi A was 87 and of *Salmonella* Typhi was 323. (Figure 2).

### Incidence and Seasonality of Paratyphoid and Typhoid Fever

**Figure 3:**
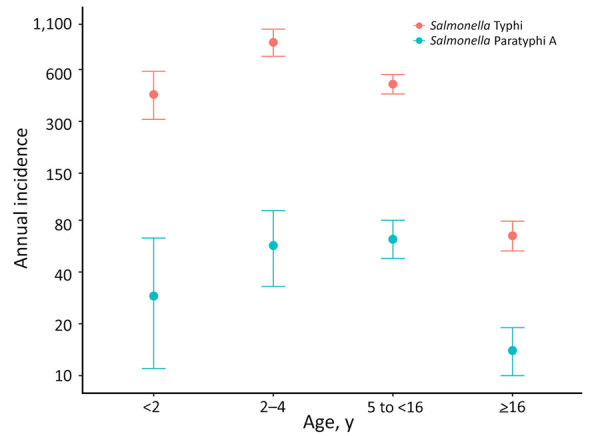
Annual incidence rates of *Salmonella* *enterica* serovars Paratyphi A and Typhi, stratified by age at follow-up, from the dynamic cohort in study of comparative epidemiology of *Salmonella* Paratyphi A and *Salmonella* Typhi causing enteric fever, Bangladesh, 2018–2020. Dots represent incidence rate, defined as no. cases/100,000 person-years. Error bars indicate 95% CIs.

The overall incidence rates (IRs) of paratyphoid for closed and dynamic cohorts were similar, with slightly different CIs: closed cohort IR 27/100,000 PY (95% CI 22–33/100,000 PY); dynamic cohort IR 27/100,000 PY (95% CI 23–32/100,000 PY). Moreover, we observed the highest incidence for both cohorts in children <16 years of age: closed cohort IR 60/100,000 PY (95% CI 46–76/100,000 PY); dynamic cohort IR 57/100,000 PY (95% CI 45–71/100,000 PY). The highest incidence for the closed cohort was among children 2–4 years of age (IR 72/100,000 PY [95% CI 41–117/100,000 PY]) followed by 5 to <16 years (IR 61/100,000 PY [95% CI 45–81/100,000 PY]), <2 years (IR 34/100,000 PY [95% CI 11–80/100,000 PY]), and ≥16 years (IR 12/100,000 PY [95% CI 8–17/100,000 PY]). The highest incidence rate for the dynamic cohort was among children 5 to <16 years of age (IR 62/100,000 PY [95% CI 48–80/100,000 PY]) followed by 2–4 years of age (IR 57/100,000 PY [95% CI 33– 91/100,000 PY]), <2 years of age (IR 29/100,000 PY [95% CI 11– 63/100,000 PY]), and >16 years of age (IR 14/100,000 PY [95% CI 10–19/100,000 PY) (Table 1; Figure 3; Appendix Table 1).

**Table 1:** Incidence rate of *Salmonella* Paratyphi A and *Salmonella* Typhi disease from a closed cohort in epidemiologic study of *Salmonella* Paratyphi A and *Salmonella* Typhi, Bangladesh, 2018–2020*

Characteristic	Total participants	*Salmonella* Paratyphi A				No. in JE arm	*Salmonella* Typhi			
		PY	No. cases	IR (95% CI)	p value†		PY	No. cases	IR (95% CI)	p value†
Overall	206,065	320,486	87	27 (22–33)	NA	103,216	160,790	323	201 (180–224)	NA
Cluster					0.461					NA
JE cluster	103,216	161,022	47	29 (22–38)		103,216	160,790	323	201 (180–224)	
Vi-TT cluster	102,849	159,464	40	25 (18–34)		NA	NA	NA	NA	
Age at baseline, y					<0.001					<0.001
<2	8,166	11,936	4	34 (11–80)		4,108	5,953	46	773 (573–1,021)	
2–4	12,494	19,545	14	72 (41–117)		6,284	9,809	87	887 (715–1,088)	
5 to <16	43,991	70,744	43	61 (45–81)		21,817	35,092	141	402 (340–472)	
>16	141,414	218,260	26	12 (8–17)		71,007	109,935	49	45 (33–58)	
<16	64,651	102,225	61	60 (46–76)	<0.001	32,209	50,854	274	539 (478–605)	<0.001
>16	141,414	218,260	26	12 (8–17)		71,007	109,935	49	45 (33–58)	
Sex					0.328					0.622
M	102,382	160,393	48	30 (22–39)		51,288	80,523	166	206 (177–239)	
F	103,683	160,093	39	24 (18–33)		51,928	80,267	157	196 (167–228)	

*IR, incidence rate, cases/100,000 persons/year; JE, Japanese encephalitis vaccine; NA, not applicable; PY, person-year; Vi-TT, Vi-tetanus toxoid conjugate vaccine. †p values determined by likelihood ratio test from the Cox-PH model.

In comparison, the overall incidence rate of typhoid for the closed cohort was 201/100,000 PY (95% CI 180–224/100,000 PY) and for the dynamic cohort 216/100,000 PY (95% CI 198–236/100,000 PY). We observed the highest incidence in children <16 years of age: closed cohort IR 539/100,000 PY (95% CI 478–605/100,000 PY) and dynamic cohort IR 558/100,000 PY (95% CI 504–616/100,000 PY). Among the children <16 years of age, the highest incidence for both cohorts was among children 2–4 years of age; closed cohort IR was 887/100,000 PY (95% CI 715–1,088/100,000 PY) and dynamic cohort IR 863/100,000 PY (95% CI 716–1,031/100,000 PY). 

**Figure 4:**
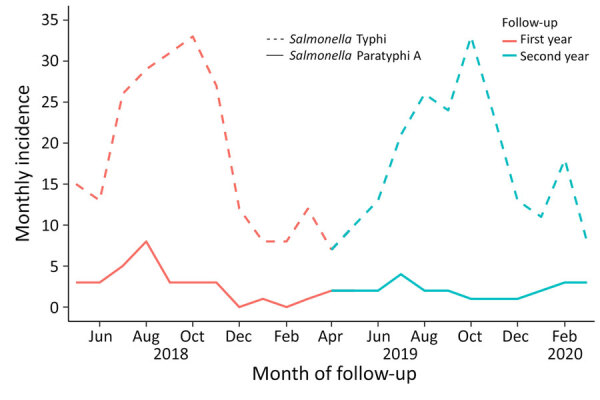
Seasonality of *Salmonella* *enterica* serovars Paratyphi A and Typhi incidence from the dynamic cohort in study of comparative epidemiology of *Salmonella* Paratyphi A and *Salmonella* Typhi causing enteric fever, Bangladesh, 2018–2020.

For the closed cohort, the incidence rate among children <2 years of age was 773/100,000 PY (95% CI 573–1,021/100,000 PY), among children 5 to <16 years was 402/100,000 PY (95% CI 340–472/100,000 PY), and among patients >16 years was 45/100,000 PY (95% CI 33–58/100,000 PY). For the dynamic cohort, the incidence rate among children <2 years of age was 430/100,000 PY (95% CI 308–586/100,000 PY), among children 5 to <16 years was 494/100,000 PY (95% CI 433–561/100,000 PY), and among patients >16 years was 65/100,000 PY (95% CI 53–79/100,000 PY). The seasonality of paratyphoid fever incidence was not apparent, whereas typhoid fever peaked in the postmonsoon period (July–August) (Figure 4; Appendix Table 2).

### Sociodemographic and WASH Characteristics Related to Paratyphoid and Typhoid Fever

The risk for paratyphoid fever was ≈3 times higher (hazard ratio 2.77 [95% CI 1.65–4.66]; p<0.001) and for typhoid fever 1.5 times higher (hazard ratio 1.55 [95% CI 1.22–1.98]; p<0.001) among members of households having no private toilet compared with members of households that had a private toilet (Tables 2, 3). Moreover, the risk for paratyphoid fever was 2 times higher (hazard ratio 2.1 [95% CI 1.19–3.72]; p = 0.011) and for typhoid 1.4 times higher (hazard ratio 1.4 [95% CI 1.07–1.82]; p = 0.013) higher among members of households having no safe source of drinking water compared with those with a safe source of drinking water. We found a significant clustering effect (ICC 0.00090 [95% CI 0.00014–0.00165]) of typhoid fever at randomized cluster levels, which was considered for the adjustment in the Cox-PH model to evaluate possible risk factors, but found no clustering effect at household cluster levels (Table 4). Moreover, we observed no significant clustering effect of paratyphoid fever at either the household or the randomized cluster levels.

**Table 2:** Associations between sociodemographic and WaSH characteristics and the occurrence of *Salmonella* Paratyphi A disease in closed cohort in epidemiologic study of *Salmonella* Paratyphi A and *Salmonella* Typhi, Bangladesh, 2018–2020*

Characteristic	No. participants	PY	No. cases	IR (95% CI)	Crude HR	p value	Adjusted HR†	p value
Religion								
Muslim	203,593	316,714	86	27 (22–33)	Referent	NA	Referent	NA
Other	2,472	3,772	1	27 (2–124)	0.98 (0.14–7.06)	0.986	1.04 (0.14–7.43)	0.973
Monthly expenditure								
Below median	100,505	153,458	46	30 (22–40)	Referent	NA	Referent	NA
Median or above	105,560	167,028	41	25 (18–33)	0.83 (0.54–1.26)	0.381	0.81 (0.53–1.24)	0.332
Toilet facility: private								
Y	85,915	141,424	18	13 (8–20)	Referent	NA	Referent	NA
N	120,150	179,062	69	39 (30–48)	2.88 (1.71–4.84)	<0.001	2.77 (1.65–4.66)	<0.001
Adult toilet: flush toilet								
Y	10,374	16,489	4	24 (8–58)	Referent	NA	Referent	NA
N	195,691	303,997	83	27 (22–34)	1.09 (0.4–2.96)	0.871	1.03 (0.38–2.81)	0.952
Child toilet: flush toilet								
Y	853	1,386	0	0 (0–178)	Referent	NA	Referent	NA
N	205,212	319,099	87	27 (22–33)	1,210,612.65 (0–∞)	0.994	1,393,520.45 (0–∞)	0.994
Source of drinking water: private tap, well, or pump; bottled water and water vendor								
Y	61,017	99,004	14	14 (8–23)	Referent	NA	Referent	NA
N	145,048	221,482	73	33 (26–41)	2.24 (1.26–3.96)	0.006	2.1 (1.19–3.72)	0.011
Treated cleaning water								
Y	889	1,387	0	0 (0–178)	Referent	NA	Referent	NA
N	205,176	319,098	87	27 (22–33)	1,210,682.57 (0–∞)	0.994	1,132,383.86 (0–∞)	0.994
Hand wash before taking meal								
Y	145,554	227,911	54	24 (18–31)	Referent	NA	Referent	NA
N	60,511	92,575	33	36 (25–49)	1.49 (0.96–2.29)	0.072	1.45 (0.94–2.24)	0.090
Hand wash after defecation								
Y	200,637	312,047	87	28 (22–34)	Referent	NA	Referent	NA
N	5,428	8,439	0	0 (0–29)	0 (0–∞)	0.994	0 (0–∞)	0.993
Handwashing water available in household								
Y	200,101	311,181	82	26 (21–33)	Referent	NA	Referent	NA
N	5,964	9,305	5	54 (20–118)	2.02 (0.82–4.99)	0.126	2.02 (0.82–4.99)	0.126
Handwashing soap available in household								
Y	202,351	314,711	87	28 (22–34)	Referent	NA	Referent	NA
N	3,714	5,775	0	0 (0–43)	0 (0–∞)	0.992	0 (0–∞)	0.992
Waste disposal place: fixed disposal								
Y	193,217	300,307	84	28 (22–34)	Referent	NA	Referent	NA
N	12,848	20,179	3	15 (4–40)	0.53 (0.17–1.67)	0.278	0.49 (0.16–1.56)	0.227
Distance to drinking water source: shorter than median distance								
Y	101,145	156,461	42	27 (20–36)	Referent	NA	Referent	NA
N	104,920	164,025	45	27 (20–36)	1.02 (0.67–1.56)	0.921	0.99 (0.65–1.51)	0.964
Water filter available in household								
Y	23,196	37,234	9	24 (12–44)	Referent	NA	Referent	NA
N	182,869	283,252	78	28 (22–34)	1.11 (0.56–2.21)	0.770	1.08 (0.54–2.15)	0.834

*HR, hazard ratio; IR, incidence rate, cases/100,000 persons/y; NA, not applicable; PY, person-years; WASH, water sanitation and hygiene. †Adjusted by age and sex.

**Table 3:** Associations between sociodemographic and WaSH characteristics and the occurrence of *Salmonella* Typhi disease in the closed cohort in epidemiologic study of *Salmonella* Paratyphi A and *Salmonella* Typhi, Bangladesh, 2018–2020*

Characteristic	Participants	PY	No. cases	IR (95% CI)	Crude HR	p value	Adjusted HR†	p value
Religion								
Muslim	102,187	159,289	323	203 (182–226)	Referent	NA	Referent	NA
Others	1,029	1,501	0	0 (0–164)	0 (0–∞)	>0.999	0 (0–∞)	>0.999
Monthly expenditure								
Below median	51,644	78,813	149	189 (160–221)	Referent	NA	Referent	NA
Median or above	51,572	81,976	174	212 (182–246)	1.15 (0.91–1.45)	0.234	1.18 (0.94–1.49)	0.160
Toilet facility: private								
Y	43,869	72,368	105	145 (119–175)	Referent	NA	Referent	NA
N	59,347	88,422	218	247 (215–281)	1.67 (1.31–2.13)	<0.001	1.55 (1.22–1.98)	<0.001
Adult toilet: flush toilet								
Y	5,002	8,055	18	223 (137–346)	Referent	NA	Referent	NA
N	98,214	152,735	305	200 (178–223)	0.89 (0.54–1.45)	0.630	0.85 (0.52–1.4)	0.533
Child toilet: flush toilet								
Y	410	689	1	145 (13–677)	Referent	NA	Referent	NA
N	102,806	160,101	322	201 (180–224)	1.44 (0.2–10.32)	0.717	2.23 (0.31–16.05)	0.425
Source of drinking water: private tap, well or pump; water vendor								
Y	32,110	52,266	78	149 (119–185)	Referent	NA	Referent	NA
N	71,106	108,524	245	226 (199–255)	1.53 (1.17–1.99)	0.002	1.4 (1.07–1.82)	0.013
Treated cleaning water								
Y	314	499	1	200 (18–934)	Referent	NA	Referent	NA
N	102,902	160,291	322	201 (180–224)	1 (0.14–7.14)	0.999	1.01 (0.14–7.23)	0.992
Hand wash before taking meal								
Y	71,253	112,173	220	196 (171–223)	Referent	NA	Referent	NA
N	31,963	48,616	103	212 (174–256)	1.07 (0.83–1.38)	0.594	1.05 (0.82–1.35)	0.707
Hand wash after defecation								
Y	100,523	156,548	314	201 (179–224)	Referent	NA	Referent	NA
N	2,693	4,241	9	212 (105–387)	0.87 (0.43–1.76)	0.698	0.81 (0.4–1.65)	0.568
Handwashing water available in household								
Y	99,578	155,046	308	199 (177–222)	Referent	NA	Referent	NA
N	3,638	5,744	15	261 (153–420)	1.31 (0.74–2.3)	0.358	1.3 (0.74–2.29)	0.363
Handwashing soap available in household								
Y	101,581	158,246	318	201 (180–224)	Referent	NA	Referent	NA
N	1,635	2,543	5	197 (75–431)	0.88 (0.34–2.28)	0.787	0.86 (0.33–2.22)	0.749
Waste disposal place: fixed disposal								
Y	97,714	152,047	307	202 (180–225)	Referent	NA	Referent	NA
N	5,502	8,743	16	183 (109–290)	0.92 (0.53–1.62)	0.782	0.85 (0.48–1.49)	0.574
Distance to drinking water source: shorter than median distance								
Y	53,075	82,428	167	203 (174–235)	Referent	NA	Referent	NA
N	50,141	78,362	156	199 (170–232)	1.01 (0.8–1.27)	0.926	0.97 (0.77–1.22)	0.821
Water filter available in household								
Y	11,804	19,125	30	157 (108–221)	Referent	NA	Referent	NA
N	91,412	141,665	293	207 (184–232)	1.31 (0.89–1.92)	0.168	1.27 (0.87–1.87)	0.218

*HR, hazard ratio; IR, incidence rate, cases/100,000 persons/year; PY, person-years; WASH, water sanitation and hygiene. †Adjusted by age and sex.

**Table 4:** Clustering of *Salmonella* Paratyphi A and *Salmonella* Typhi infection in epidemiologic study, Bangladesh, 2018–2020*

Clustering level	*Salmonella* Paratyphi A			*Salmonella* Typhi	
	No. clusters	ICC (95% CI)		No. clusters	ICC (95% CI)
Randomized clusters	150	0.00028 (−0.00013 to 0.00069)		75	0.00090 (0.00014–0.00165)
HH clusters	50,688	0.00000012 (−1 to 1)		25,478	0.00000010 (−1 to 1)

*HH, household; ICC, intracluster correlation

### Clinical Characteristics of Paratyphoid and Typhoid Fever

We found no significant differences in clinical features between paratyphoid and typhoid patients, even when stratified by age groups (Table 5). The mean temperature recorded at enrollment for paratyphoid patients was 37.8°C and for typhoid patients, 37.9°C. We observed high fever, defined as an axillary temperature of >40°C, in 2/121 (1.7%) of paratyphoid and 3/483 (0.6%) of typhoid patients when they sought care. Five (4.1%) of 121 paratyphoid patients and 24 (5.0%) of 483 typhoid patients were admitted to a hospital. Of those, 35/121 (28.9%) paratyphoid and 109/483 (22.6%) typhoid patients had a history of antimicrobial drug intake during the 2 weeks before seeking care at the medical facility. Gastrointestinal (abdominal pain, nausea, vomiting, diarrhea) and lower respiratory tract (cough, wheeze, tachypnea) symptoms were equally common among the paratyphoid and typhoid patients. Among paratyphoid patients, 29/121 (24.0%) experienced gastrointestinal symptoms and 33/121 (27.3%) lower-respiratory symptoms; among typhoid patients, 147/483 (30.4%) experienced gastrointestinal symptoms and 130/483 (26.9%) lower-respiratory symptoms. All patients recovered, and none experienced severe complications such as intestinal hemorrhage, perforation, or encephalopathy.

**Table 5:** Clinical findings of *Salmonella* Paratyphi A and *Salmonella* Typhi by age group from cases detected in the dynamic cohort in epidemiologic study, Bangladesh, 2018–2020*

Clinical finding	Paratyphoid cases from all clusters, n = 121	Typhoid cases from JE clusters, n = 483	p value
Overall	121 (100)	483 (100)	
Mean body temperature, °C (±SD)	37.8 (±0.95)	37.9 (±0.93)	0.165†
High fever, ≥40°C	2 (1.7)	3 (0.6)	0.263‡
Antibiotic taken in past 2 weeks	35 (28.9)	109 (22.6)	0.177
Admitted to hospital	5 (4.1)	24 (5.0)	0.883
Median duration of hospitalization, d (IQR)	6 (5–6)	9.5 (5–13)	0.139§
Gastrointestinal tract diseases¶	29 (24.0)	147 (30.4)	0.198
Lower respiratory tract diseases#	33 (27.3)	130 (26.9)	>0.999
Upper respiratory tract diseases**	12 (9.9)	67 (13.9)	0.316
Neurologic diseases‡‡	13 (10.7)	39 (8.1)	0.450
Rash/new lesion	1 (0.8)	3 (0.6)	>0.999‡
<5 years of age	22 (18.2)	189 (39.1)	
Mean body temperature, °C (±SD)	37.8 (±1.04)	37.9 (±0.92)	0.677†
High fever, ≥40°C	2 (9.1)	0 (0.0)	0.010‡
Antibiotic taken in past 2 weeks	8 (36.4)	39 (20.6)	0.159
Admitted to hospital	1 (4.5)	8 (4.2)	>0.999‡
Median duration of hospitalization, d (IQR)	4 (4–4)	10.5 (10–13)	0.241§
Gastrointestinal tract diseases¶	4 (18.2)	59 (31.2)	0.324‡
Lower respiratory tract diseases#	8 (36.4)	59 (31.2)	0.803
Upper respiratory tract diseases**	5 (22.7)	33 (17.5)	0.753
Neurologic diseases‡‡	1 (4.5)	5 (2.6)	0.488‡
Rash/new lesion	0 (0.0)	0 (0.0)	>0.999‡
>5 years of age	99 (81.8)	294 (60.9)	
Mean body temperature, °C (±SD)	37.8 (±0.93)	37.9 (±0.95)	0.221†
High fever (≥40°C)	0 (0.0)	3 (1.0)	0.575‡
Antibiotic taken in last 2 weeks	27 (27.3)	70 (23.8)	0.578
Admitted to hospital	4 (4.0)	16 (5.4)	0.792‡
Median duration of hospitalization, d (IQR)	6 (6–6)	8 (5–13)	0.669§
Gastrointestinal tract diseases¶	25 (25.3)	88 (29.9)	0.446
Lower respiratory tract diseases#	25 (25.3)	71 (24.1)	0.932
Upper respiratory tract diseases**	7 (7.1)	34 (11.6)	0.282
Neurologic diseases‡‡	12 (12.1)	34 (11.6)	>0.999
Rash/new lesion	1 (1.0)	3 (1.0)	>0.999‡

*Values are no. (%) except as indicated. p values derived from χ^2^ test except as indicated. †By *t*-test for mean difference. ‡By Fisher exact test. §By Mann-Whitney U test. ¶Gastrointestinal includes any of the following: abdominal pain, nausea, vomiting, diarrhea. #Lower respiratory tract includes any of the following: cough, wheeze, tachypnea, respiratory distress. **Upper respiratory tract includes any of the following: sore throat, coryza, ear pain, enlarged tonsils, painful/swollen lymph nodes in head/neck, stridor. ‡‡Neurological includes any of the following: headache, photophobia, neck stiffness, seizure.

### AMR Patterns

One (0.8%) of the 121 *Salmonella* Paratyphi A isolates was MDR; the rest were susceptible to the first-line antimicrobial drugs ampicillin, chloramphenicol, and trimethoprim/sulfamethoxazole. In contrast, 97/480 (20.2%) *Salmonella* Typhi isolates were MDR (p<0.001 for the difference in occurrence between *Salmonella* Paratyphi A and *Salmonella* Typhi). Of note, azithromycin resistance was significantly more frequent (p<0.001) among *Salmonella* Paratyphi A isolates (14/121 [11.6%]) than among *Salmonella* Typhi isolates (9/483 [1.9%]). However, we did not observe resistance to any third-generation cephalosporin tested for either pathogen. Almost all *Salmonella* Paratyphi A (119/121 [98.3%]) and *Salmonella* Typhi (456/483 [94.4%]) isolates were resistant to nalidixic acid and had intermediate resistance to ciprofloxacin (119/121 [98.3%] *Salmonella* Paratyphi A and 411/483 [85.1%] *Salmonella* Typhi isolates) (Table 6).

**Table 6:** Antimicrobial resistance of *Salmonella* Paratyphi A and *Salmonella* Typhi isolates from the dynamic cohort in epidemiologic study, Bangladesh, 2018–2020*

Drug	No. resistant (%)		p value
	*Salmonella *Paratyphi A isolates from all clusters, n = 121	*Salmonella *Typhi isolates from JE clusters, n = 483	
Ampicillin	1/121 (0.8)	194/482 (40.2)	<0.001
Chloramphenicol	2/121 (1.7)	120/479 (25.1)	<0.001
Trimethoprim/sulfamethoxazole	1/120 (0.8)	122/482 (25.3)	<0.001
Amoxiclav	0/121 (0.0)	38/482 (7.9)	<0.001
Azithromycin	14/121 (11.6)	9/483 (1.9)	<0.001
Cefixime	0/121 (0.0)	0/483 (0.0)	>0.999
Ceftriaxone	0/121 (0.0)	0/483 (0.0)	>0.999
Gentamicin	0/121 (0.0)	2/482 (0.4)	>0.999
Meropenem	0/121 (0.0)	0/482 (0.0)	>0.999
Ciprofloxacin†	1/121 (0.8)	57/483 (11.8)	<0.001
Nalidixic acid	119/121 (98.3)	456/483 (94.4)	0.229
MDR for ampicillin, chloramphenicol, trimethoprim/sulfamethoxazole	1/121 (0.8)	97/480 (20.2)	<0.001

*p values determined by Fisher exact test. MDR, multidrug resistance. †Intermediate result for *Salmonella* Paratyphi A, 119/121 (98.3%); for *Salmonella *Typhi, 411/483 (85.1%).

## Discussion

In an urban slum population in Dhaka, Bangladesh, we identified a moderate incidence of paratyphoid fever (defined as 10–100/100,000 PY) and a high incidence of typhoid fever (defined as >100/100,000 PY) (*26*). The lack of private toilets and safe drinking water was one of the key risk factors for both diseases. Our findings highlight a high prevalence of both diseases in the population predominantly affecting the same age group and sharing similar risk factors; integrated control and preventive measures might include the use of bivalent vaccines for both typhoid and paratyphoid fevers and improved WASH. We observed a higher burden of MDR in *Salmonella* Typhi, whereas *Salmonella* Paratyphi A remained largely susceptible to former first-line drugs but exhibited higher azithromycin resistance. Both pathogens exhibited intermediate resistance to ciprofloxacin.

This study is one of the few detailed comparative epidemiologic investigations on paratyphoid and typhoid fever in Bangladesh undertaken concurrently for both diseases. We found a higher incidence of typhoid fever than paratyphoid fever, consistent with previous studies (*1*,*27*,*28*). The Surveillance for Enteric Fever in Asia Project in 2016–2019 (*27*) estimated a crude incidence of typhoid fever as 103/100,000 PY and of paratyphoid fever as 16/100,000 PY in Bangladesh. A population-based study under the STRATAA (Strategic Typhoid Alliance across Africa and Asia) consortium also conducted in Dhaka in 2016–2018 (*28*) showed crude incidences of typhoid as 161/100,000 PY and paratyphoid as 42/100,000 PY. However, during the TCV efficacy study, the incidence of typhoid fever in Nepal (342/100,000 PY) (*29*) and Bangladesh (213/100,000 PY) (*20*) was higher than in Malawi (182.7/100,000 PY) (*30*) in the control group. 

Previous studies from Dhaka noted that some environmental factors, such as the preceding rainy season, higher temperature, higher rainfall, or water level of nearby water sources, were associated with a higher incidence of typhoid fever (*31*,*32*). Estimating the typhoid prevalence is challenging, given that the factors influencing blood culture positivity can vary widely. Existing studies have shown a strong correlation between enteric fever incidence and poor sanitation and limited access to clean drinking water (*33*,*34*) which aligns with our WASH risk factor analysis. Although establishing optimal WASH infrastructure is resource heavy, our findings suggest that public health interventions to improve private toilets and safe drinking water sources should be considered as a near-term investment for tackling both diseases.

The STRATAA study showed the highest reported typhoid incidence in Kathmandu and Dhaka among the 5–9-year age group (*28*), whereas in this study, we found that children <16 years of age had the highest risk for enteric fever. This finding suggests a bivalent vaccine against both pathogens could be targeted for children <16 years of age. Among children <16 years of age, the highest incidence for both diseases was among children 2–4 years of age, emphasizing that immunization programs with a bivalent vaccine should prioritize infants <2 years of age, as has been done with the implementation of TCV in typhoid-endemic countries (*20*,*29*,*30*).

AMR constitutes a compelling driver for the development of new-generation bivalent vaccines as well as improved WASH interventions. Although first-line antimicrobial drugs remain effective for treating paratyphoid fever in our setting, MDR *Salmonella* Typhi is highly prevalent. Because the 2 diseases are difficult to distinguish based on clinical features, the selection of appropriate first-line antimicrobial drugs is difficult in many settings (*35*). Moreover, nalidixic acid resistance and ciprofloxacin intermediate resistance in both pathogens, as well as the substantial level of resistance to azithromycin in *Salmonella* Paratyphi A, indicate that empiric use of these drugs for treating enteric fever might not be effective in most patients (*2*,*9*–*11*,*36*). Furthermore, third-generation cephalosporin-resistant *Salmonella* Typhi has been reported in Pakistan (*37*). Our study found both organisms to be sensitive to cephalosporin, but Pakistan’s rapid cephalosporin resistance spread signals a need for vigilant monitoring and prudent antimicrobial use.

TCVs have been approved for implementation in the Expanded Program of Immunization of Bangladesh. However, TCVs do not provide cross-protection against *Salmonella* Paratyphi A. After a period of TCV implementation, the prevalence of paratyphoid fever may increase, perhaps because of strain replacement, as was observed in China with the Vi-polysaccharide typhoid vaccine (*38*). In aggregate, our findings thus strongly support preventing both diseases by devising effective bivalent vaccines and WASH interventions (*19*,*39*,*40*).

The first limitation of our study was its 2-year duration; seasonality analysis would be strengthened with a longer surveillance period. Second, some cases might have been missed because we enrolled a fraction of eligible participants from the 8 healthcare facilities. Third, the available clinical data were categorized according to systems, not on the basis of individual symptoms or signs, which limited our ability to identify distinguishing clinical features. Fourth, the WASH data was based on a simple questionnaire and could have missed important information. Last, our study was limited to an urban setting in Dhaka, so the findings might not be generalizable to rural areas or other geographic locations. Despite all those limitations, the strength of this study was the prospective, comprehensive, and concurrent surveillance of a large study population for both paratyphoid and typhoid fever, together with repeated censuses, which enabled us to analyze the dynamic cohort in a highly mobile population along with closed cohort from the baseline study population.

In conclusion, we found that, although the incidence of *Salmonella* Typhi in the study area in Dhaka is greater than that of *Salmonella* Paratyphi A, the effect of *Salmonella* Paratyphi A is not negligible, especially in children. Vaccination with a bivalent vaccine should be programmatically feasible given the similar age-specific patterns of incidence, and the similarities of WASH factors associated with the risk for each pathogen suggest that simple WASH interventions might be effective against both pathogens.

Appendix Additional information about study of comparative epidemiology of *Salmonella* Paratyphi A and *Salmonella* Typhi causing enteric fever, Bangladesh, 2018–2020
