# Supplementary material for: Comparative Epidemiology of Salmonella enterica Serovers Paratyphi A and Typhi Causing Enteric Fever, Bangladesh, 2018–2020
Source: Emerg Infect Dis. 2025 Oct;31(10):1922–34. doi: 10.3201/eid3110.241601 (PMC12483018; doi:10.3201/eid3110.241601)
Supplement: Appendix — Additional information about study of comparative epidemiology of Salmonella Paratyphi A and Salmonella Typhi causing enteric fever, Bangladesh, 2018–2020 [file 24-1601-Techapp-s1.pdf]

Article DOI: https://doi.org/10.3201/eid3110.241601

EID cannot ensure accessibility for supplementary materials supplied by authors. Readers who have difficulty accessing supplementary content should contact the authors for assistance.

Comparative Epidemiology of *Salmonella* Paratyphi A and *Salmonella* Typhi Causing Enteric Fever, Bangladesh, 2018–2020

Appendix

Appendix Table 1. Overall and age (age at follow-up) stratified incidence rate of *Salmonella* Paratyphi A and *Salmonella* Typhi from the dynamic cohort*

		Salmonella Paratyphi A				*Salmonella* Typhi		
Characteristic	Total no. participants	Person-years	No. cases	IR* (95% CI)	No. participants only in JE arm	Person-years	No. cases	IR (95% CI)
Overall	326,794	445,668	121	27 (23, 32)	163,371	223,342	483	216 (198, 236)
Age at follow-up								
<2 years	23,404	17,282	5	29 (11–63)	11,630	8,603	37	430 (308–586)
2 to 4 years	32,112	26,545	15	57 (33–91)	16,019	13,331	115	863 (716–1031)
5 to <16 years	84,963	93,298	58	62 (48–80)	42,093	46,550	230	494 (433–561)
>16 years	265,929	308,549	43	14 (10–19)	132,695	154,860	101	65 (53–79)
<16 years	140,479	137,125	78	57 (45–71)	69,742	68,484	382	558 (504–616)
>16 years	265,929	308,549	43	14 (10–19)	132,695	154,860	101	65 (53–79)

*IR, Incidence rate per 100,000 persons per year; JE, Japanese encephalitis vaccine.

Appendix Table 2. Seasonality of *Salmonella* Paratyphi A and *Salmonella* Typhi incidence from dynamic cohort*

		Salmonella Paratyphi A				*Salmonella* Typhi		
Month	Total no. participants	Person-months	No. cases	IR	No. participants only in JE arm	Person-months	No. cases	IR
May 2018	208,291	195,760	6	3	104,441	98,082	15	15
Jun 2018	237,851	218,387	7	3	119,350	109,311	14	13
Jul 2018	235,272	219,657	11	5	117,289	109,694	28	26
Aug 2018	232,767	219,991	17	8	116,013	109,814	32	29
Sep 2018	232,986	222,444	7	3	116,260	111,119	34	31
Oct 2018	232,855	224,255	7	3	116,580	112,232	37	33
Nov 2018	232,504	224,525	7	3	116,352	112,341	30	27
Dec 2018	241,796	225,479	1	0	120,247	112,828	13	12
Jan 2019	259,943	235,900	3	1	130,188	118,342	9	8
Feb 2019	246,245	234,847	1	0	123,574	117,706	10	8
Mar 2019	246,837	237,409	2	1	123,922	119,062	14	12
Apr 2019	246,095	237,527	4	2	123,453	119,140	8	7
May 2019	246,022	237,905	4	2	123,396	119,336	12	10
Jun 2019	263,053	238,574	4	2	131,668	119,851	16	13
Jul 2019	250,515	236,332	9	4	125,923	118,628	25	21
Aug 2019	247,637	235,135	4	2	124,235	117,991	31	26
Sep 2019	246,395	236,558	5	2	123,369	118,524	29	24
Oct 2019	245,410	237,130	2	1	122,941	118,811	39	33
Nov 2019	245,596	237,019	2	1	123,030	118,754	27	23
Dec 2019	248,931	237,157	2	1	124,564	118,831	15	13
Jan 2020	254,124	233,813	5	2	127,433	117,473	13	11
Feb 2020	241,712	229,220	6	3	121,123	115,261	21	18
Mar 2020	240,179	105,559	3	3	120,858	53,038	4	8

*IR, Incidence rate per 100,000 persons per month; JE, Japanese encephalitis vaccine.
